# Supplementary material for: Altered Mitochondrial Opa1-Related Fusion in Mouse Promotes Endothelial Cell Dysfunction and Atherosclerosis
Source: Antioxidants (Basel). 2022 May 28;11(6):1078. doi: 10.3390/antiox11061078 (PMC9219969; doi:10.3390/antiox11061078)
Supplement: Supplementary file 1 [file antioxidants-11-01078-s001.zip › antioxidants-1751265-supplementary.pdf]

Altered mitochondrial Opa1-related fusion in mouse promotes endothelial cell dysfunction and atherosclerosis

Ahmad Chehaitly, Anne-Laure Guihot, Coralyne Proux, Linda Grimaud, Jade Aurrière, Benoit Legouriellec, Jordan Rivron, Emilie Vessieres, Clément Tétaud, Antonio Zorzano , Vincent Procaccio, Françoise Joubeau, Pascal Reynier, Guy Lenaers, Laurent Loufrani, Daniel Henrion

Supplemental figures

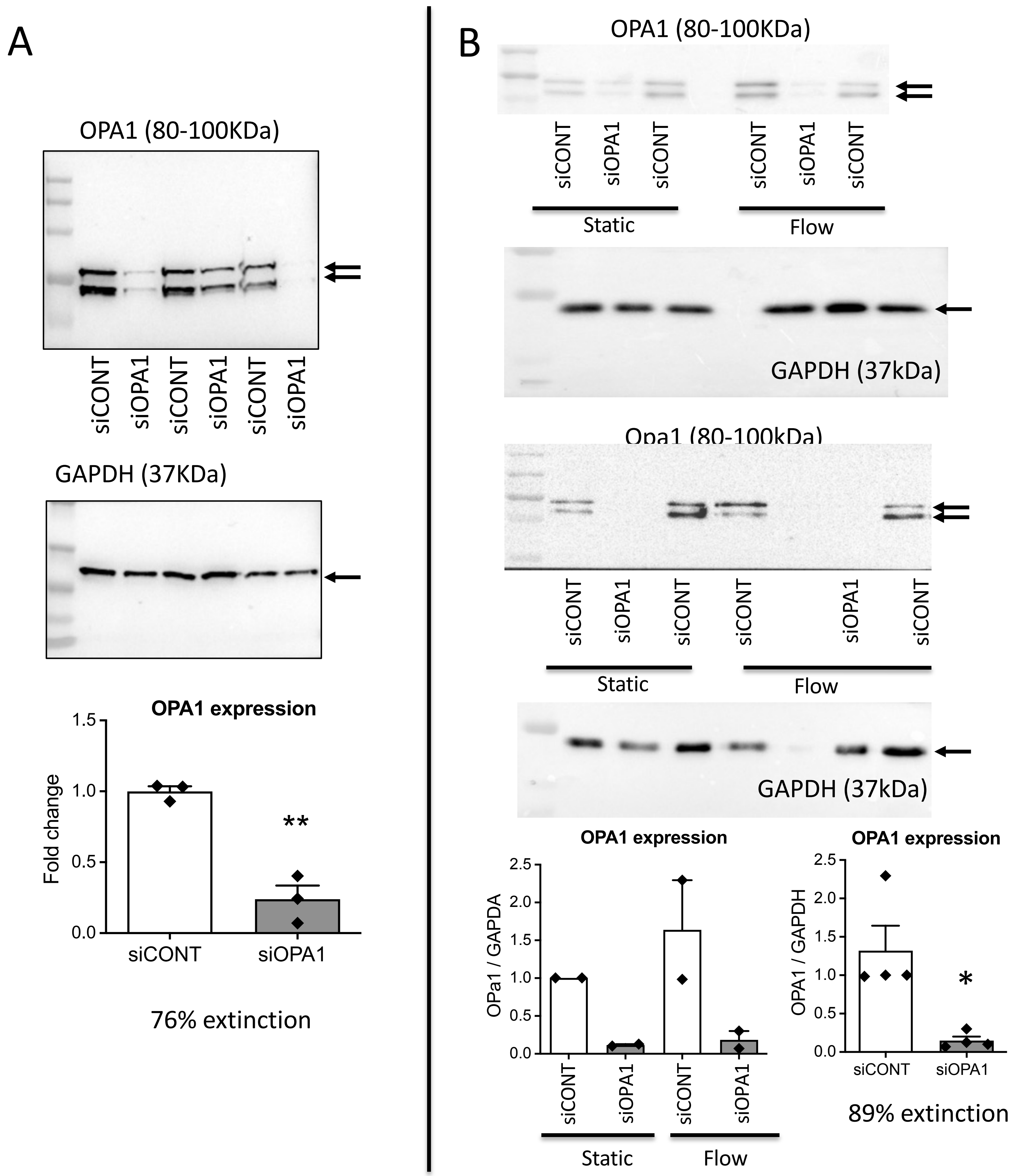

Figure S1: Validation of OPA1 silencing in HUVECs.
Protein expression level of OPA1 was determined in HUVECs with Opa1 silencing (siOPA1) or not (siCONT) for 72 hours in static conditions (A, corresponding to figure 1 A to E) or submitted to laminar flow for 24h following the 72h (total: 96h in the presence of siOPA1) (B, corresponding to Figure 1 F to M). Means ± the SEM are shown (n = 2 or 3 independent experiments).

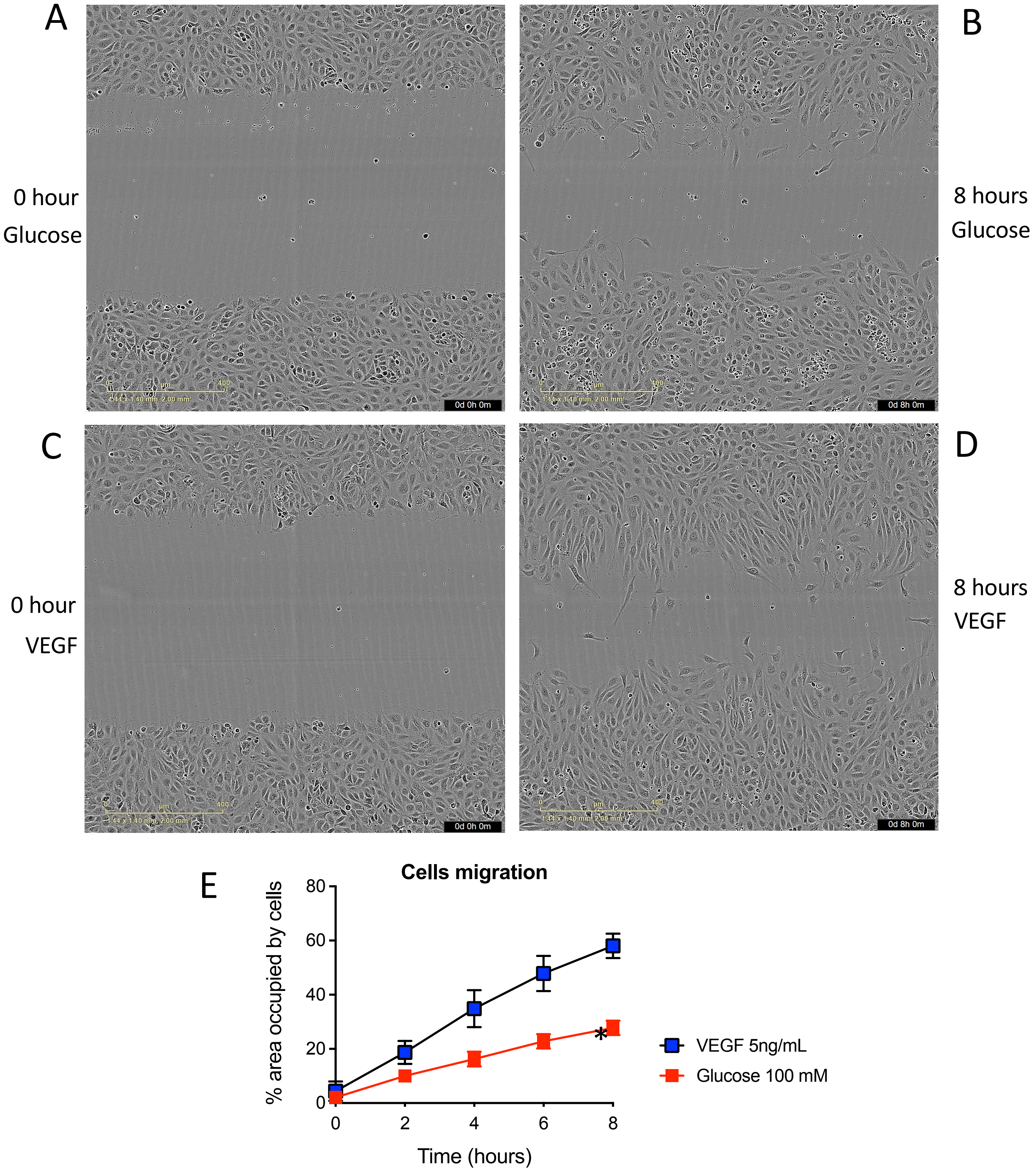

Figure S2: **Positive and negative controls of ECs migration**
Endothelial cells (HUVECs) migration was measured in the presence of Glucose 100mM (A,B) as negative control or in the presence of VEGF (5 ng/mL) as positive control (C,D) during 8 hours. Data is expressed as the area occupied by cells after each time point (E). Means ± SEM are shown (n = 3 independent experiments per group).
*p=0.0170 (interaction: p<0.0001), 2-way ANOVA for repeated measurements.

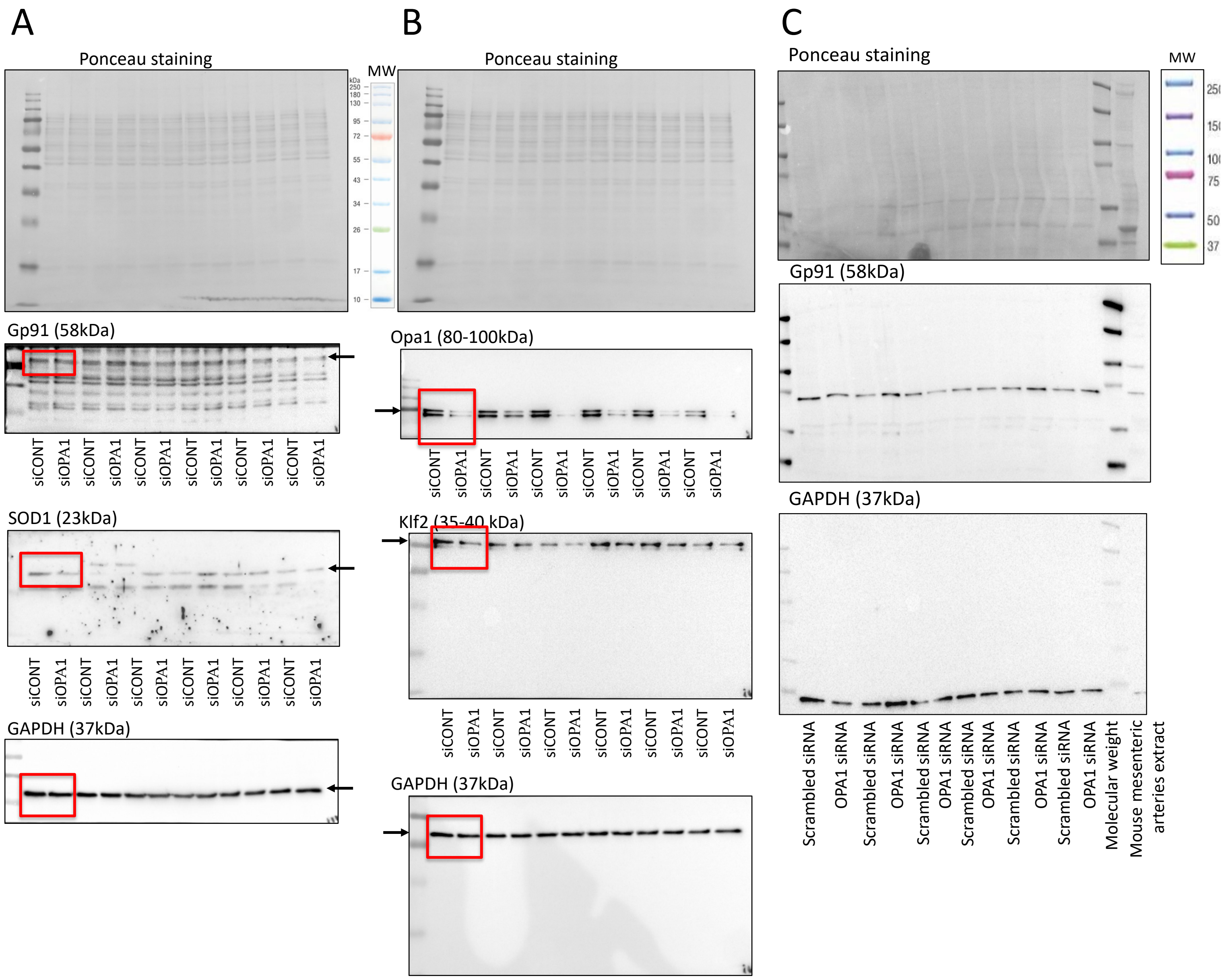

Figure S3: Whole blots corresponding to the graphs shown in figure 2 A, C, F and G).

A and B: HUVECs were submitted with OPA1 silencing (siOPA1) or not (siCONT) for 72 hours in and then submitted to laminar flow for 24h (total: 96h in the presence of siOPA1).
Red triangle: blots shown on figure 2

C: second set of experiments to confirm the data show in figure 2G. The western blot for gp91 was performed with another antibody than in A (reference: CAB11966, AssayGenie, Dublin, Ireland).

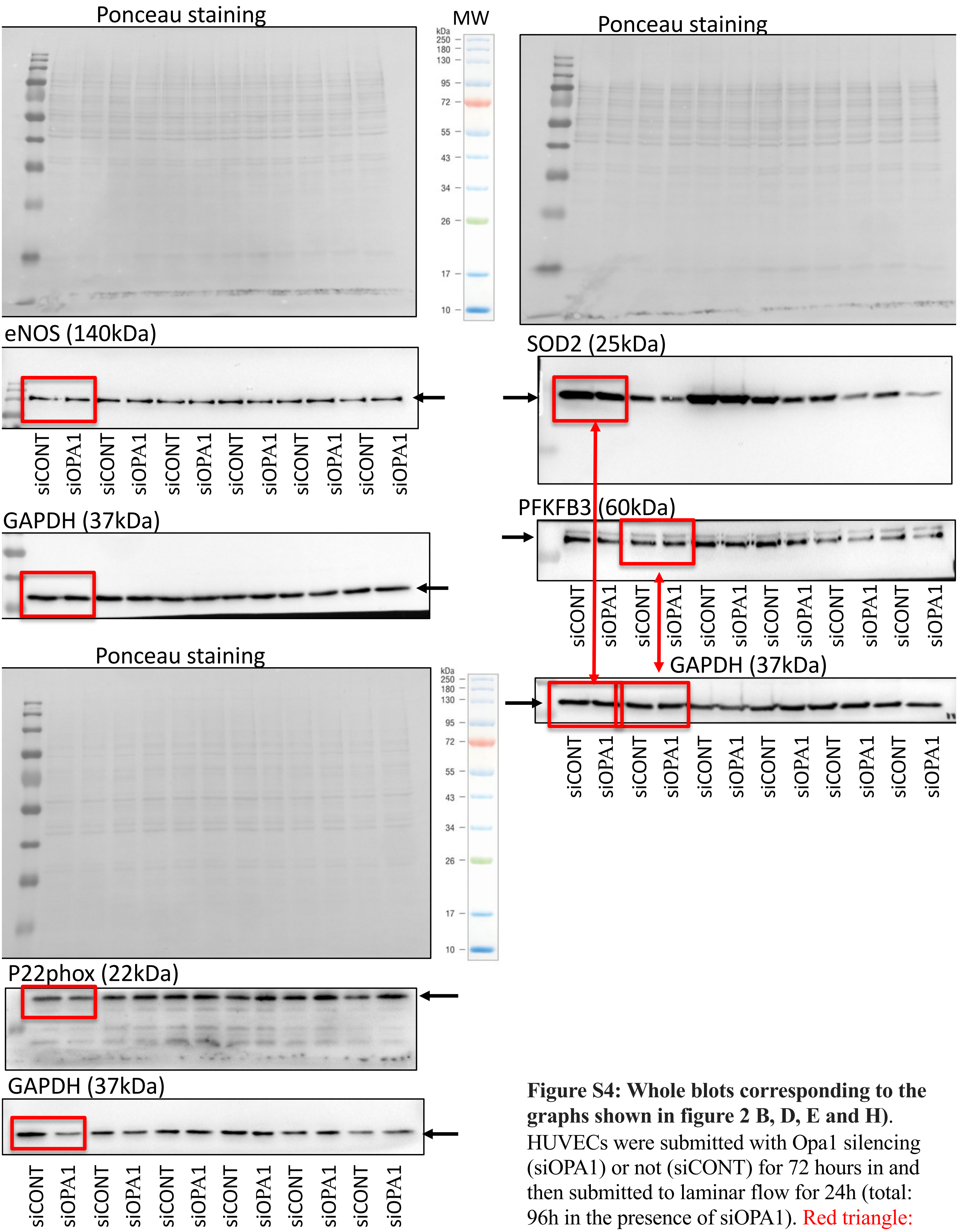

Figure S4: Whole blots corresponding to the graphs shown in figure 2 B, D, E and H). HUVECs were submitted with Opa1 silencing (siOPA1) or not (siCONT) for 72 hours in and then submitted to laminar flow for 24h (total: 96h in the presence of siOPA1). Red triangle: blots shown on figure 2

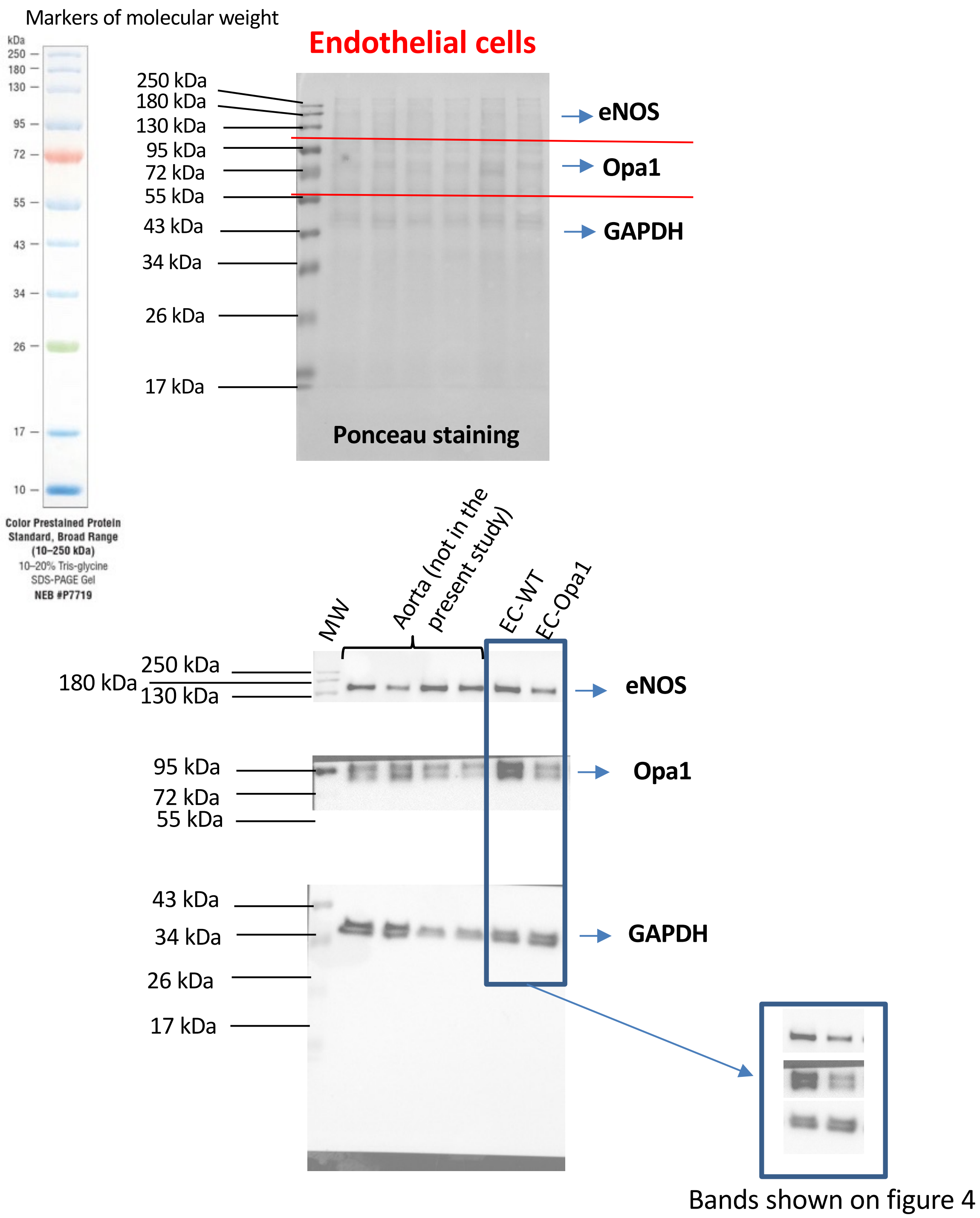

Figure S5: Validation of Opa1 extinction in mesenteric artery endothelial cells isolated from EC-Opa1 or EC-WT mice.

Protein expression level of Opa1, eNOS and GAPDH was determined in mesenteric artery endothelial cells isolated from EC-Opa1 or EC-WT mice.

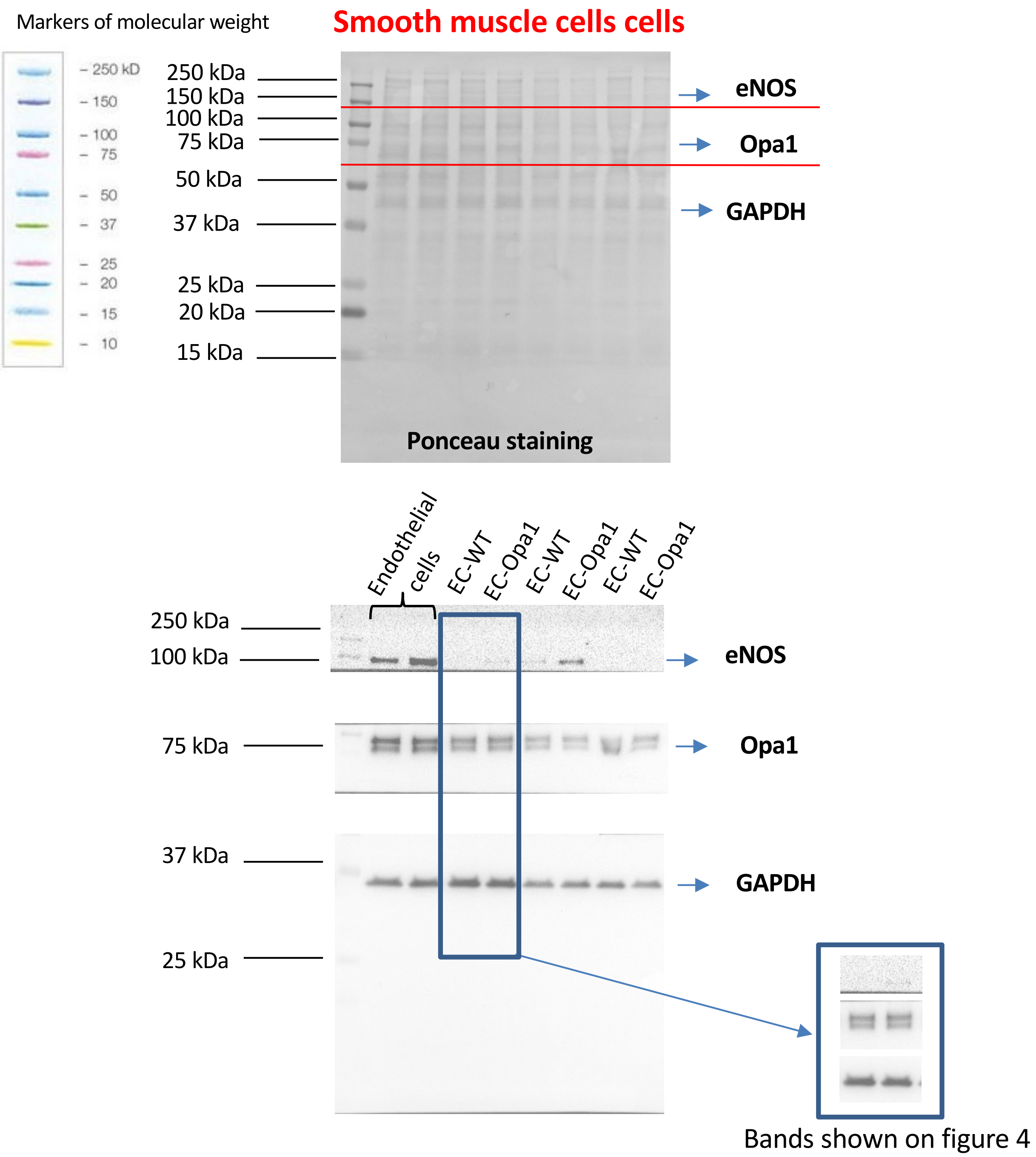

Figure S6: Validation of Opa1 extinction in mesenteric artery smooth muscle cells isolated from EC-Opa1 or EC-WT mice.

Protein expression level of Opa1, eNOS and GAPDH was determined in mesenteric artery endothelial cells isolated from EC-Opa1 or EC-WT mice. Endothelial cells were loaded in 2 bands as a positive control for eNOS.
